# Associations between modifiable risk factors and limitation in activities of daily living among individuals aged ≥ 45 years: Evidence from the China health and retirement longitudinal study (CHARLS)

**DOI:** 10.3934/publichealth.2025050

**Published:** 2025-10-13

**Authors:** Yaheng Li, Jian Gao, Wenzhu Song, Xiaolin Liang, Xinhao He, Fuliang Yi, Wenhao Song, Dongliang Yin

**Affiliations:** 1 School of Traditional Chinese Medicine, Jiangxi University of Chinese Medicine, Nanchang, Jiangxi, 330004, China; 2 Department of Clinical Laboratory, Heping Branch, Shanxi Provincial People's Hospital, Taiyuan, Shanxi, 030012, China; 3 Department of Anesthesiology, Xi'an Eighth Hospital, Xi'an, Shaanxi, 710061, China; 4 School of Public Health, Shanxi Medical University, Taiyuan, Shanxi, 030001, China; 5 Department of Geriatrics, Zhongshan Hospital of Traditional Chinese Medicine, Zhongshan, Guangdong, 528401, China; 6 School of Sports Medicine and Rehabilitation, Beijing Sport University, Beijing 100084, China; 7 Department of Public Health, Zigong Fourth People's Hospital, Zigong, Sichuan, 643000, China

**Keywords:** modifiable risk factors, physically functional disability, cross-sectional study, logistic regression, population aging

## Abstract

**Background:**

With aging populations, a growing number of older people are subjected to limitations in activities of daily living (ADL), causing a tremendous burden and challenges to patients' quality of life and policymakers. Investigating modifiable risk factors for ADL remains an important project to help lower its risk. This study aimed to explore associations between the modifiable risk factors and ADL using national-scale data from the China Health and Retirement Longitudinal Study (CHARLS).

**Methods:**

Data were downloaded from CHARLS 2020, a population-based longitudinal survey. We included modifiable risk variables and ADL index, i.e., basic ADL (BALD) and instrumental ADL (IADL). Afterward, variables were included in the logistic regression model construction. Also, propensity score matching (PSM) was employed to validate the findings. Finally, we tried to discuss the associations between different age groups.

**Results:**

A total of 12,248 participants were included in this study, comprising 5799 women (47.35%) and 6449 men (52.65%). The median age was 62 (55–69) years. Among the participants, 2055 (16.78%) had limitations in BADL, and 1838 (15.01%) had limitations in IADL. Logistic regression demonstrated that exercise significantly reduced the risk of BADL and IADL impairment (BADL: *OR* = 0.70, 95% *CI*: 0.59–0.83; IADL: *OR* = 0.65, 95% *CI*: 0.55–0.78; *P* < 0.001). Similarly, better cognitive ability was associated with a lower risk of impairment (BADL: *OR* = 0.68, 95% *CI*: 0.61–0.75; IADL: *OR* = 0.60, 95% *CI*: 0.54–0.67; *P* < 0.001). Adequate sleep duration (6–8 hours) also significantly reduced the likelihood of functional decline (BADL: *OR* = 0.49, 95% *CI*: 0.45–0.55; IADL: *OR* = 0.48, 95% *CI*: 0.43–0.53; *P* < 0.001). In contrast, depression symptoms and chronic diseases significantly increased the risk of both BADL and IADL impairment. Besides, PSM showed similar findings, and the risk of ADL increased with age.

**Conclusions:**

Modifiable risk factors, such as exercise, cognitive ability, depression symptoms, chronic diseases, social activities, and sleeping duration, were significantly associated with ADL. Besides, as age increases, the impact of various modifiable risk factors on ADL becomes more evident, emphasizing special care for older populations and offering scientific ideas for policymakers.

## 1. Introduction

Over the past decades, the world has witnessed a continuing increase in an aging population. By 2050, it is projected that the older population will reach 400 million aged ≥ 65 years, registering a stunning 26.9% of the globe, and 150 million aged ≥ 80 years [1], triggering critical challenges for healthcare professionals and policymakers [2]. Another major consequence of this shift in age structure is the surging number of those with physical functional disabilities (PFD). Currently, PFD poses a pressing threat to improved quality of life in later years and places a heavy burden on a sustainable health and social care system.

According to the International Classification of Functioning, Disability and Health (ICF), disability emphasizes the combined effects of impairments, activity limitations, or participation restrictions. PFD has specifically assessed limitations in activities, particularly in the execution of basic activities of daily living (BADL), such as dressing, bathing, and eating, and instrumental activities of daily living (IADL), including tasks like housework, meal preparation, medication management, financial management, and telephone use [3]. Previous studies showed that BADL and IADL could represent independent indicators for effectively predicting PFD among the older population [4]. The World Health Organization (WHO) reported that 1.3 billion people, 16% of the global population, currently live with PFD, largely due to longer life expectancy [5]. China, home to the world's largest older population, holds a disability prevalence rate of 26% [6] among the older population. A study has estimated that older adults will live an average of 7.44 years with disability by 2030, increasing to 11.45 years by 2050 [7]. Obviously, PFD has emerged as a global public health issue.

With limited effective strategies available, lowering the risk of ADL limitation has become a more crucial focus or priority. Even if better approaches are accessible, risk mitigation may persist as a significant one to lower the number of those affected [8]. Undoubtedly, drug administration serves as an effective approach for some non-communicable diseases, which may help explain the limited physical movement, but it is accompanied by some side effects [9], and no specific medications are guaranteed to modify limitations in ADL. As such, investigation and management of risk factors remain of prime importance in the effort to navigate PFD and are essential components of the “Healthy China 2030 Planning”.

Recently, some researchers have also focused on risk factor discussion for limitations in ADL. An example is Wang et al., who demonstrated that a sleep duration of more than 12 hours may be associated with an increased risk of ADL disability in the oldest-old individuals; the optimal sleep time for this population appears to be 8–10 hours [10]. Another case concerns Alexandre et al., whose study suggested that dynapenic abdominal obesity is a key risk factor for impairment in physical function among older adults [11]. Besides, dysphoria and anhedonia are viewed as risk factors for disability or mortality in older populations [12]. However, previous studies are constrained to limited sample sizes, inadequate analysis of modifiable risk factors, and a lack of focus on different age groups. Consequently, the evidence remains inconclusive.

Notably, a study identified three key strategies to reduce care dependency and improve the quality of life of the older population: improving educational attainment, creating age-friendly living environments, and expanding healthcare access. Yet, these three approaches are subject to government actions [13], and cannot be achieved by individuals themselves by modifying their daily habits. Presently, one of the goals of the Healthy China action plan to promote the health of the older adults is to reduce disability incidence [14]. If each older individual were to make small improvements in their daily activities, it would reduce reliance on government support and significantly enhance their physical capacities.

To handle the gap between modifiable risk factors and urgent needs for effective interventions, this study aimed to explore associations between modifiable risk factors and limitations in ADL, using national-scale data from the China Health and Retirement Longitudinal Study (CHARLS). These findings could inform a scientific, older people–targeted intervention approach for policymakers and act as a contributor to the achievement of “Health China 2030”.

## 2. Methods

### 2.1. Data sources and participants

Data was downloaded from The China Health and Retirement Longitudinal Study (CHARLS) 2020 (http://charls.pku.edu.cn/en/n). CHARLS is the first nationally representative population survey in China targeting individuals aged 45 and older, providing a high-quality publicly available dataset [15]. It is a longitudinal survey of residents of the Chinese mainland, aged 45 and above, covering 150 districts and 450 villages/urban communities across the country, involving 19,752 people in 10,257 families, comprehensively reflecting the collective situation of China's middle-aged and older population.

The fifth wave of the survey included a total of 19,395 individuals. For this study, participants who did not meet the inclusion criteria were excluded. Specifically, 128 individuals under 45 years old, 47 with missing ADL data, 1085 with a sleep duration of fewer than 3 hours or more than 20 hours, 5380 with missing cognitive ability data, 504 with missing depression symptoms, and 3 with missing residence information were excluded. After these exclusions, a total of 12,248 individuals were included in the study. All rounds of the CHARLS survey were approved by the Ethics Committee of Peking University (IRB00001052–11015), and all participants offered informed consent. All methods were performed in accordance with the relevant guidelines and regulations.

### 2.2. Variable collection

This study included demographic indicators such as marital status, age, gender, education levels, and residence. The independent variables are sleeping duration, exercise, alcohol consumption, smoking, social activities, and chronic diseases, including depressive symptoms and cognitive ability. The dependent variables are limitations in basic activities of daily living (BADL) and instrumental activities of daily living (IADL).

### 2.3. Variable definitions

#### 2.3.1. Covariate variables

Age is categorized into three groups: 45–59 years, 60–69 years, and 70 years and older. Gender included male and female. Residence was referred to the Chinese Hukou system, a means of population registration, not unlike a census crossed with an in-country passport. In this study, it was defined as rural and non-rural areas. Marital status was defined as “married”, which included “married with spouse present” and “married but not living with spouse temporarily for reasons such as work”, and “others”, which included “separated, no longer living together as a spouse”, “divorced”, “widowed”, and “never married”. Educational levels were categorized into four groups: “no formal education”, which includes those who are illiterate, did not finish primary school, or received education through Sishu or homeschooling; “elementary education”, which refers to those who completed elementary school; “secondary education”, which includes those who finished middle school; and “higher education”, which encompasses high school, vocational school, two- or three-year colleges/associate degrees, four-year colleges/bachelor's degrees, master's degrees, and doctoral degrees/Ph.D.

#### 2.3.2. Independent variables

Current drinking, current smoking, and exercise were defined as “yes” or “no”. Sleeping durations were categorized into <6 hours, 7–8 hours, and >8 hours.

As for chronic diseases, they were collected by asking “Have you been diagnosed with the following conditions by a doctor”, including hypertension, diabetes, cancer, lung disease, heart disease, stroke, mental illness, arthritis, dyslipidemia, liver disease, kidney disease, stomach disease, asthma, and memory disorders (including Alzheimer's disease and brain atrophy). A response of “1” indicates the presence of at least one chronic disease, while “0” indicates the absence. If any of these conditions are present, it is defined as “yes”; otherwise, it is defined as “no”.

Social activities included seven types of social activities: visiting friends or neighbors; playing mahjong, chess, or cards, or going to a community activity room; providing unpaid help to relatives, friends, or neighbors not living with you; dancing, exercising, or practicing Qigong in parks or other venues; participating in activities organized by social groups; engaging in volunteer or charitable activities; and attending school or training courses. These activities were defined as either “yes” or “no” based on the question in the questionnaire: “Have you participated in any of the following social activities in the past month?” where “1” indicates “yes” and “0” indicates “no”. Participation in at least one of the seven social activities was defined as engaging in social activities; otherwise, it was defined as not participating.

The assessment of depressive symptoms utilized the 10-item short version of the Center for Epidemiologic Studies Depression Scale (CES–D–10) [16]. The scale includes the following items: (1) I was bothered by things that usually don't bother me; (2) I had trouble keeping my mind on what I was doing; (3) I felt depressed; (4) I felt that everything I did was an effort; (5) I felt hopeful about the future; (6) I felt fearful; (7) My sleep was restless; (8) I was happy; (9) I felt lonely; (10) I felt I could not get going. Each item is rated on a 4-point scale: “Rarely or none of the time (<1 day)”; “Some or a little of the time (1–2 days)”; “Occasionally or a moderate amount of the time (3–4 days)”; and “Most or all of the time” (5–7 days). These responses are scored as 0, 1, 2, and 3 points, respectively, except for items 5 and 8, which are reverse-scored. The total score ranges from 0 to 30, with a score of 10 or higher indicating the presence of depressive symptoms.

Cognitive ability, as a form of human capital, includes two components: mental status and episodic memory. Mental status is evaluated through tests of date orientation, calculation, and drawing abilities, with a maximum score of 11 points. Episodic memory is assessed through word recall, with a maximum score of 10 points. The total cognitive ability score ranges from 0 to 21, with higher scores indicating better cognitive performance [17]. Based on the median score, individuals are classified into low and high cognitive ability groups.

#### 2.3.3. Dependent variables

Activities of daily living (ADL) include dressing, bathing, eating, getting in and out of bed, using the toilet, and controlling bowel and bladder function. Instrumental activities of daily living (IADL) include housekeeping, cooking, shopping, managing finances, and taking medications [18]. The response options are “no difficulty”, “difficulty but can be completed”, “difficulty and requires assistance”, and “unable to complete”. The total scores for ADL and IADL are calculated separately. If there is difficulty in completing any of these tasks, it is determined that the individual has ADL limitation.

### 2.4. Statistical analysis

All statistical analyses were performed by R (version 4.3.1). Continuous data are represented as mean (*SD*), median (*P_25_, P_75_*). Accordingly, the t-test and Wilcoxon rank-sum test were employed to assess differences in continuous variables, and the *χ^2^* test was utilized for comparisons between the normal group and limited groups for BADL and IADL. To identify any possible association between the modified factors and ADL, univariate and multivariable logistic regression model analysis were used to determine the odds ratio (*OR*) and 95% confidence interval (*CI*), which were achieved using the “autoReg” package. For multivariable regression analysis, the independent association was examined using two logistic regression models.

Model 1 (the crude model) was not adjusted for any covariates. Model 2 (full model) was adjusted to control for age, gender, education levels, marital status, and residence. Subgroup analyses were also conducted, with age groups being stratified. Next, to mitigate potential baseline confounders, a propensity score matching (PSM) analysis was conducted, matching those with impaired ADL with those not. Balanced variables for matching included age, gender, education levels, marital status, and residence. The “nonrandom” package in R was utilized for inter-cohort propensity score matching. This process utilized the nearest neighbor algorithm with a 1:1 matching ratio and a caliper value set to 0.02. To assess the balance of covariates before and after matching, the standardized mean difference (SMD), with an absolute value of the mean difference less than 0.1, was considered a successful matching [19]. A significance level of *P* < 0.05 indicated statistically significant differences, with all *P*-values being two-sided.

## 3. Results

### 3.1. Baseline characteristics

A total of 12,248 participants were included in this study, comprising 5799 women (47.35%) and 6449 men (52.65%). The median age was 62 (55–69) years. Among the participants, 2055 (16.78%) had limitations in BADL, and 1838 (15.01%) had limitations in IADL. Females made up 56.84% and 58.05% of the BADL and IADL limitation groups, respectively. Marital status, residence, and educational level were significantly associated with BADL and IADL disability (*P* < 0.001). Additionally, the differences in BADL and IADL disability among the modifiable risk factors were also statistically significant (*P* < 0.001) (Table 1). Age, marital status, educational level, exercise, current drinking, current smoking, sleeping duration, cognitive ability, and social activities, as well as BADL and IADL, differed significantly between genders (*P* < 0.05). In contrast, residence and chronic diseases did not show significant differences between genders (*P* > 0.05), as shown in Table 2.

**Table 1.** Baseline characteristics of the study population in different ADL statuses.

Characteristic	Overall (12,248)	BADL			IADL		
		Normal (10,193)	Limited (2055)	*P*	Normal (10,410)	Limited (1838)	*P*
Age, *n* (%), years				<0.001			<0.001
45–59	5756 (47.00)	5108 (50.11)	648 (31.53)		5159 (49.56)	597 (32.48)	
60–69	4049 (33.06)	3268 (32.06)	781 (38.00)		3385 (32.52)	664 (36.13)	
≥70	2443 (19.95)	1817 (17.83)	626 (30.46)		1866 (17.93)	577 (31.39)	
Gender				<0.001			<0.001
Women	5799 (47.35)	4631 (45.43)	1168 (56.84)		4732 (45.46)	1067 (58.05)	
Men	6449 (52.65)	5562 (54.57)	887 (43.16)		5678 (54.54)	771 (41.95)	
Marital status, *n* (%)				<0.001			<0.001
No	1394 (11.38)	1073 (10.53)	321 (15.62)		1098 (10.55)	296 (16.10)	
Yes	10,854 (88.62)	9120 (89.47)	1734 (84.38)		9312 (89.45)	1542 (83.90)	
Residence, *n* (%)				<0.001			<0.001
Non-rural	3602 (29.41)	3113 (30.54)	489 (23.80)		3154 (30.30)	448 (24.37)	
Rural	8646 (70.59)	7080 (69.46)	1566 (76.20)		7256 (69.70)	1390 (75.63)	
Educational levels, *n* (%)				<0.001			<0.001
No formal education	3523 (28.76)	2699 (26.48)	824 (40.10)		2767 (26.58)	756 (41.13)	
Elementary education	3139 (25.63)	2592 (25.43)	547 (26.62)		2631 (25.27)	508 (27.64)	
Secondary education	3503 (28.60)	3040 (29.82)	463 (22.53)		3090 (29.68)	413 (22.47)	
Higher education	2083 (17.01)	1862 (18.27)	221 (10.75)		1922 (18.46)	161 (8.76)	
Exercise, *n* (%)				<0.001			<0.001
No	858 (7.01)	653 (6.41)	205 (9.98)		664 (6.38)	194 (10.55)	
Yes	11,390 (92.99)	9540 (93.59)	1850 (90.02)		9746 (93.62)	1644 (89.45)	
Current drinking, *n* (%)				<0.001			<0.001
No	7236 (59.08)	5875 (57.64)	1361 (66.23)		5956 (57.21)	1280 (69.64)	
Yes	5012 (40.92)	4318 (42.36)	694 (33.77)		4454 (42.79)	558 (30.36)	
Current smoking, *n* (%)				<0.001			<0.001
No	8773 (71.63)	7185 (70.49)	1588 (77.27)		7348 (70.59)	1425 (77.53)	
Yes	3475 (28.37)	3008 (29.51)	467 (22.73)		3062 (29.41)	413 (22.47)	
Chronic diseases, *n* (%)				<0.001			<0.001
No	2488 (20.31)	2356 (23.11)	132 (6.42)		2377 (22.83)	111 (6.04)	
Yes	9760 (79.69)	7837 (76.89)	1923 (93.58)		8033 (77.17)	1727 (93.96)	
Sleeping duration, *n* (%) (h)				<0.001			<0.001
<6	3935 (32.13)	2952 (28.96)	983 (47.83)		3034 (29.15)	901 (49.02)	
7–8	7622 (62.23)	6659 (65.33)	963 (46.86)		6780 (65.13)	842 (45.81)	
>8	691 (5.64)	582 (5.71)	109 (5.30)		596 (5.73)	95 (5.17)	
Cognitive ability, *n* (%)				<0.001			<0.001
Low	5989 (48.90)	4704 (46.15)	1285 (62.53)		4789 (46.00)	1200 (65.29)	
High	6259 (51.10)	5489 (53.85)	770 (37.47)		5621 (54.00)	638 (34.71)	
Depression symptoms, *n* (%)				<0.001			<0.001
No	8176 (66.75)	7346 (72.07)	830 (40.39)		7498 (72.03)	678 (36.89)	
Yes	4072 (33.25)	2847 (27.93)	1225 (59.61)		2912 (27.97)	1160 (63.11)	
Social activities, *n* (%)				<0.001			<0.001
No	5771 (47.12)	4711 (46.22)	1060 (51.58)		4807 (46.18)	964 (52.45)	
Yes	6477 (52.88)	5482 (53.78)	995 (48.42)		5603 (53.82)	874 (47.55)	

Note: BADL, basic activity of daily living; IADL, instrumental activity of daily living.

**Table 2.** Baseline characteristics of the study population by gender.

Variables	Overall (12,248)	Women (5799)	Men (6449)	*P*
Age, *n* (%), years				<0.001
45–59	5756 (47.00)	3021 (52.10)	2735 (42.41)	
60–69	4049 (33.06)	1805 (31.13)	2244 (34.80)	
≥70	2443 (19.95)	973 (16.78)	1470 (22.79)	
Marital status, *n* (%)				<0.001
No	1394 (11.38)	866 (14.93)	528 (8.19)	
Yes	10,854 (88.62)	4933 (85.07)	5921 (91.81)	
Residence, *n* (%)				0.196
Non-rural	3602 (29.41)	1738 (29.97)	1864 (28.90)	
Rural	8646 (70.59)	4061 (70.03)	4585 (71.10)	
Educational levels, *n* (%)				<0.001
No formal education	3523 (28.76)	2185 (37.68)	1338 (20.75)	
Elementary education	3139 (25.63)	1422 (24.52)	1717 (26.62)	
Secondary education	3503 (28.60)	1423 (24.54)	2080 (32.25)	
Higher education	2083 (17.01)	769 (13.26)	1314 (20.38)	
Exercise, *n* (%)				0.032
No	858 (7.01)	376 (6.48)	482 (7.47)	
Yes	11,390 (92.99)	5423 (93.52)	5967 (92.53)	
Current drinking, *n* (%)				<0.001
No	7236 (59.08)	4705 (81.13)	2531 (39.25)	
Yes	5012 (40.92)	1094 (18.87)	3918 (60.75)	
Current smoking, *n* (%)				<0.001
No	8773 (71.63)	5572 (96.09)	3201 (49.64)	
Yes	3475 (28.37)	227 (3.91)	3248 (50.36)	
Chronic diseases, *n* (%)				0.345
No	2488 (20.31)	1157 (19.95)	1331 (20.64)	
Yes	9760 (79.69)	4642 (80.05)	5118 (79.36)	
Sleeping duration, *n* (%), (h)				<0.001
<6	3935 (32.13)	2096 (36.14)	1839 (28.52)	
7–8	7622 (62.23)	3423 (59.03)	4199 (65.11)	
>8	691 (5.64)	280 (4.83)	411 (6.37)	
Cognitive ability, *n* (%)				0.002
Low	5989 (48.90)	2922 (50.39)	3067 (47.56)	
High	6259 (51.10)	2877 (49.61)	3382 (52.44)	
Depression symptoms, *n* (%)				<0.001
No	8176 (66.75)	3452 (59.53)	4724 (73.25)	
Yes	4072 (33.25)	2347 (40.47)	1725 (26.75)	
Social activities, *n* (%)				0.009
No	5771 (47.12)	2660 (45.87)	3111 (48.24)	
Yes	6477 (52.88)	3139 (54.13)	3338 (51.76)	
BADL, *n* (%)				<0.001
Normal	10193 (83.22)	4631 (79.86)	5562 (86.25)	
Limited	2055 (16.78)	1168 (20.14)	887 (13.75)	
IADL, *n* (%)				<0.001
Normal	10,410 (84.99)	4732 (81.60)	5678 (88.04)	
Limited	1838 (15.01)	1067 (18.40)	771 (11.96)	

Note: BADL, basic activity of daily living; IADL, instrumental activity of daily living.

### 3.2. Characteristics of modified risk factors in different ADL statuses

Regarding exercise, individuals who do not engage in physical activity represent 9.98% of the BADL limitation group and 10.55% of the IADL limitation group. In terms of current alcohol consumption, non-drinkers account for a higher proportion in the limitation groups, with 66.23% for BADL and 69.64% for IADL. When it comes to smoking, 22.73% of the BADL limitation group and 22.47% of the IADL limitation group are smokers. Concerning cognitive function, individuals with low cognitive levels make up 62.53% of the BADL limitation group and 65.29% of the IADL limitation group. For depression, the proportion of individuals experiencing depressive symptoms is significantly higher in the limited groups, at 59.61% for BADL and 63.11% for IADL. Regarding chronic diseases, a vast majority of those in the limitation groups have at least one chronic condition, with 93.58% for BADL and 93.96% for IADL. For sleep duration, individuals who sleep less than 6 hours have the highest proportions in the limitation groups, at 47.83% for BADL and 49.02% for IADL, while those sleeping 7–8 hours represent 46.86% and 45.81% of these groups, respectively. Finally, individuals who do not participate in social activities account for 51.58% and 52.45% of the BADL and IADL limitation groups, respectively (Table 3).

**Table 3.** Characteristics of modified risk factors in different ADL groups.

Variables	BADL		IADL	
	Normal (10,193)	Limited (2055)	Normal (10,410)	Limited (1838)
Exercise, *n* (%)				
No	653 (6.41)	205 (9.98)	664 (6.38)	194 (10.55)
Yes	9540 (93.59)	1850 (90.02)	9746 (93.62)	1644 (89.45)
Current drinking, *n* (%)				
No	5875 (57.64)	1361 (66.23)	5956 (57.21)	1280 (69.64)
Yes	4318 (42.36)	694 (33.77)	4454 (42.79)	558 (30.36)
Current smoking, *n* (%)				
No	7185 (70.49)	1588 (77.27)	7348 (70.59)	1425 (77.53)
Yes	3008 (29.51)	467 (22.73)	3062 (29.41)	413 (22.47)
Cognitive ability, *n* (%)				
Low	4704 (46.15)	1285 (62.53)	4789 (46.00)	1200 (65.29)
High	5489 (53.85)	770 (37.47)	5621 (54.00)	638 (34.71)
Depression symptoms, *n* (%)				
No	7346 (72.07)	830 (40.39)	7498 (72.03)	678 (36.89)
Yes	2847 (27.93)	1225 (59.61)	2912 (27.97)	1160 (63.11)
Chronic diseases, *n* (%)				
No	2356 (23.11)	132 (6.42)	2377 (22.83)	111 (6.04)
Yes	7837 (76.89)	1923 (93.58)	8033 (77.17)	1727 (93.96)
Sleeping duration, *n* (%) (h)				
<6	2952 (28.96)	983 (47.83)	3034 (29.15)	901 (49.02)
7–8	6659 (65.33)	963 (46.86)	6780 (65.13)	842 (45.81)
>8	582 (5.71)	109 (5.30)	596 (5.73)	95 (5.17)
Social activities, *n* (%)				
No	4711 (46.22)	1060 (51.58)	4807 (46.18)	964 (52.45)
Yes	5482 (53.78)	995 (48.42)	5603 (53.82)	874 (47.55)

### 3.3. Association between modifiable risk factors and limitations in ADL

Four multivariable logistic regression models were employed to examine the association between modifiable risk factors and functional disabilities. Due to the large sample size, all potential risk factors were included for analysis in this study. The dependent variables represent BADL and IADL, and the modifiable risk factors were independent variables. Variables and their assignments are available in Table 4.

For BADL limitation, in the crude model, without covariate adjustments, exercise, current drinking, current smoking, better cognition, social activities, and sleeping 6–8 hours or more than 8 hours were significantly negatively associated with BADL limitation, while depression symptoms and chronic diseases increased the risk (all *P* < 0.001). These associations remained significant in model 2 after adjusting for various covariates (Table 4).

For IADL limitation, the crude model revealed significant negative associations with exercise, current drinking, current smoking, better cognition, social activities, and sleeping 6–8 hours or more than 8 hours. Conversely, depression and chronic diseases were associated with an increased risk of IADL limitation (all *P* < 0.001). After adjusting for covariates in model 2, these associations remained statistically significant (Table 5).

### 3.4. Propensity score matching

To further highlight the robust associations between modifiable risk factors and PFD, propensity score matching (PSM) was employed to address covariate imbalances between the normal and limited groups for both BADL and IADL. The covariates considered included marital status, age, sex, residence, and educational level. As shown in Supplementary Table S1 and Supplementary Table S2, before PSM, the differences between the normal and limited groups were statistically significant (*P* < 0.05). However, after PSM, these differences were no longer statistically significant (*P* > 0.05). Additionally, the standardized mean differences (SMD) after PSM were all <0.1, indicating effective matching.

After propensity score matching (PSM), a logistic regression model was used to examine associations with the matched data. For both BADL and IADL limitations, exercise, current drinking, better cognition, social activities, and sleep duration of 6–8 hours or ≥8 hours were significantly associated with a lower risk of limitations. Conversely, chronic disease significantly increased the risk for both BADL and IADL limitations. Current smoking did not have a significant effect on either BADL or IADL limitations (Table 6).

**Table 4.** Variables and their assignments.

Variables	Assignments
Exercise (x_1_)	No = 0*; Yes = 1
Current drinking (x_2_)	No = 0*; Yes = 1
Current smoking (x_3_)	No = 0*; Yes = 1
Cognitive ability (x_4_)	Low = 0*; High = 1
Depression symptoms (x_5_)	No = 0*; Yes = 1
Chronic diseases (x_6_)	No = 0*; Yes = 1
Social Activities (x_7_)	No = 0*; Yes = 1
Sleeping Duration (h) (x_8_)	<6 = 1*; 6–8 = 2; >8 = 3
BADL (y_1_)	Normal = 0*; Limited = 1
IADL (y_2_)	Normal = 0*; Limited = 1

Note: *Reference.

**Table 5.** Association between modifiable risk factors and BADL and IADL.

Models Variables	BADL		IADL	
	Crude model	Model 2	Crude model	Model 2
	*OR* (95% *CI*)	*OR* (95% *CI*)	*OR* (95% *CI*)	*OR* (95% *CI*)
Exercise	0.62 (0.52–0.73)***	0.70 (0.59–0.83)***	0.58 (0.49–0.68)***	0.65 (0.55–0.78)***
Current drinking	0.69 (0.63–0.77)***	0.95 (0.85–1.06)	0.58 (0.52–0.65)***	0.78 (0.69–0.88)**
Current smoking	0.70 (0.63–0.78)***	0.89 (0.78–1.02)	0.70 (0.62–0.78)***	0.91 (0.79–1.05)
Cognitive ability	0.51 (0.47–0.57)***	0.68 (0.61–0.75)***	0.45 (0.41–0.50)***	0.60 (0.54–0.67)***
Depression symptoms	3.81 (3.45–4.20)***	3.37 (3.05–3.73)***	4.41 (3.97–4.89)***	3.88 (3.48–4.32)***
Chronic diseases	4.38 (3.66–5.28)***	3.84 (3.19–4.62)***	4.60 (3.80–5.64)***	4.04 (3.33–4.96)***
Social activities	0.81 (0.73–0.89)***	0.89 (0.81–0.98)*	0.78 (0.70–0.86)***	0.86 (0.77–0.95)**
Sleeping duration (6–8 h)	0.43 (0.39–0.48)***	0.49 (0.45–0.55)***	0.42 (0.38–0.46)***	0.48 (0.43–0.53)***
Sleeping duration (≥8 h)	0.56 (0.45–0.70)***	0.52 (0.42–0.65)***	0.54 (0.42–0.67)***	0.50 (0.40–0.63)***

Note: Crude model: No covariate was adjusted. Model 2 (full model): Age, gender, education levels, marital status, and residence were adjusted. ***P < 0.001; **P < 0.01, *P < 0.05.

**Table 6.** Association between modifiable risk factors and physical functional disability.

Variables	BADL		IADL	
	*OR* (95% *CI*)	*P*	*OR* (95% *CI*)	*P*
Exercise	0.65 (0.52–0.81)	<0.001	0.59 (0.47–0.75)	<0.001
Current drinking	0.86 (0.75–0.97)	0.017	0.74 (0.65–0.85)	<0.001
Current smoking	0.88 (0.77–1.02)	0.092	0.89 (0.77–1.04)	0.150
Cognitive ability	0.65 (0.57–0.73)	<0.001	0.58 (0.51–0.66)	<0.001
Depression symptoms	3.28 (2.88–3.73)	<0.001	3.70 (3.23–4.25)	<0.001
Chronic disease	4.92 (4.03–6.05)	<0.001	4.87 (3.92–6.09)	<0.001
Social activities	0.85 (0.75–0.96)	0.007	0.81 (0.71–0.92)	0.001
Sleeping duration (6–8 h)	0.48 (0.42–0.54)	<0.001	0.47 (0.41–0.54)	<0.001
Sleeping duration (≥8 h)	0.55 (0.42–0.73)	<0.001	0.51 (0.38–0.69)	<0.001

### 3.5. Association between modifiable risk factors and limitations in ADL in different age groups

To further explore the strong associations, different age groups were considered for analysis. Age at 45–59 years was taken as a reference, and we then assessed the impact of modifiable risk factors on limitations in ADL in different age groups. Notably, the modifiable risk factors remain robustly statistically significant in different age groups (Table 7).

One of the most significant risk factors, particularly in the ≥70 age group, was individuals who did not engage in exercise; these had a much higher risk of both BADL and IADL limitations. Depression was strongly associated with functional limitations. In the ≥70 age group, the risk of IADL limitation was notably high, highlighting the substantial impact of mental health on daily functioning. Lower cognitive ability was also a significant predictor, with older adults (≥70 years) having a higher risk of BADL and IADL limitations.

Additional factors such as smoking, alcohol consumption, sleep duration, and social activities were also associated with risk for functional limitations. For instance, non-participation in social activities and inadequate sleep were linked to increased risks, particularly in the older age group. However, the effects of these factors, while significant, were comparatively less obvious.

**Table 7.** Association between modifiable risk factors and ADL limitations in different age groups

Variables	Age (years)	BADL		IADL	
		*OR* (95% *CI*)	*P*	*OR* (95% *CI*)	*P*
Age	69–70	1.88 (1.68–2.11)	<0.001	1.7 (1.51–1.91)	<0.001
	≥70	2.72 (2.4–3.07)	<0.001	2.67 (2.36–3.03)	<0.001
Exercise					
No	69–70	1.76 (1.18–2.62)	0.006	2.21 (1.45–3.36)	<0.001
	≥70	3.31 (2.22–4.94)	<0.001	4.24 (2.78–6.46)	<0.001
Yes	69–70	1.89 (1.68–2.12)	<0.001	1.65 (1.46–1.87)	<0.001
	≥70	2.62 (2.3–2.98)	<0.001	2.5 (2.19–2.86)	<0.001
Current drinking					
No	69–70	1.82 (1.58–2.09)	<0.001	1.59 (1.38–1.84)	<0.001
	≥70	2.56 (2.2–2.98)	<0.001	2.51 (2.15–2.93)	<0.001
Yes	69–70	1.94 (1.6–2.34)	<0.001	1.82 (1.48–2.24)	<0.001
	≥70	2.85 (2.31–3.51)	<0.001	2.77 (2.21–3.47)	<0.001
Current smoking					
No	69–70	1.9 (1.67–2.16)	<0.001	1.64 (1.43–1.87)	<0.001
	≥70	2.54 (2.21–2.92)	<0.001	2.54 (2.2–2.94)	<0.001
Yes	69–70	1.94 (1.53–2.47)	<0.001	2.01 (1.57–2.58)	<0.001
	≥70	3.37 (2.61–4.35)	<0.001	3.18 (2.43–4.16)	<0.001
Cognitive ability					
Low	69–70	1.57 (1.35–1.83)	<0.001	1.47 (1.26–1.72)	<0.001
	≥70	2.40 (2.05–2.81)	<0.001	2.24 (1.9–2.63)	<0.001
High	69–70	2.02 (1.70–2.40)	<0.001	1.65 (1.36–1.99)	<0.001
	≥70	2.43 (1.99–2.98)	<0.001	2.55 (2.07–3.15)	<0.001
Depression symptoms					
No	69–70	2.00 (1.68–2.37)	<0.001	1.78 (1.47–2.16)	<0.001
	≥70	2.87 (2.39–3.46)	<0.001	3.3 (2.71–4.02)	<0.001
Yes	69–70	1.78 (1.52–2.08)	<0.001	1.61 (1.37–1.89)	<0.001
	≥70	2.45 (2.06–2.92)	<0.001	2.14 (1.8–2.55)	<0.001
Chronic diseases					
No	69–70	1.69 (1.13–2.51)	0.01	1.52 (0.99–2.33)	0.054
	≥70	2.29 (1.42–3.71)	0.001	1.54 (0.87–2.72)	0.135
Yes	69–70	1.71 (1.51–1.92)	<0.001	1.53 (1.35–1.74)	<0.001
	≥70	2.38 (2.09–2.7)	<0.001	2.38 (2.08–2.71)	<0.001
Social activities					
No	69–70	1.79 (1.53–2.11)	<0.001	1.51 (1.28–1.79)	<0.001
	≥70	2.67 (2.25–3.17)	<0.001	2.49 (2.09–2.96)	<0.001
Yes	69–70	1.94 (1.66–2.28)	<0.001	1.86 (1.57–2.2)	<0.001
	≥70	2.67 (2.24–3.19)	<0.001	2.77 (2.3–3.33)	<0.001
Sleeping duration (h)					
<6	69–70	1.56 (1.31–1.85)	<0.001	1.57 (1.31–1.88)	<0.001
	≥70	2.44 (2.03–2.93)	<0.001	2.61 (2.16–3.16)	<0.001
6–8	69–70	1.98 (1.69–2.32)	<0.001	1.65 (1.4–1.95)	<0.001
	≥70	2.60 (2.18–3.1)	<0.001	2.41 (2–2.89)	<0.001
≥8	69–70	2.51 (1.45–4.36)	0.001	1.63 (0.93–2.85)	0.089
	≥70	3.13 (1.78–5.47)	<0.001	2.42 (1.39–4.22)	0.002

Note: The age group of 45–59 years was used for reference.

## 4. Discussion

This study examines the associations between modifiable risk factors, such as sleeping duration, social activities, depression symptoms, and limitations in ADL, using a nationally representative sample of older adults from the fifth wave of the national CHARLS 2020 survey. The findings were strongly validated using PSM, and as age increases, the impact of various modifiable risk factors on limitations in ADL becomes more evident, indicating that the risk of limitations in ADL increases with age. This study offers a new perspective on the prevention and management of functional disability and holds valuable insights for advancing the “Healthy China 2030” initiative.

Previous studies also tried to explore the risk factors for limitations in ADL [20],[21]. Comparatively, our study focuses on various specific modifiable risk factors, allowing for more targeted insights. There is growing evidence that physical activity improves cognitive function and mental health. One study suggested that rural dwellers may be engaged in more physical activities, such as ploughing and harvesting, which can be useful in maintaining physical functioning [18]. Meanwhile, according to activity theory, individuals require a sense of fulfilment and belonging within their social networks. A Korean study showed that the greater the intensity of recreational exercise among older adults, the better their IADL [22]. Older adults are inclined to partake in activities that promote their social integration and facilitate adaptation to their changing environment [18]. Our results were consistent with them, showing that exercise (*OR* = 0.62, 95% *CI*: 0.52–0.73) was significantly negatively associated with BADL and IADL limitations. Not participating in an exercise program is associated with BADL and IADL limitations.

Normally, alcohol consumption is known to be harmful to health, and drinking alcohol increases the risk of cancer and heart disease [23]. However, a study demonstrated that compared to non-drinkers, light drinkers had a 21% lower risk of dementia, and moderate drinkers had a 17% lower risk of dementia; however, higher alcohol consumption increased the risk of dementia, with heavy drinkers having an 8% increased risk of dementia [24], which may impact functional ability. Another study showed that alcohol consumption may be associated with better ADLs in Chinese older adults [25]. A cross-sectional study has shown that past smoking is positively associated with changes in ADL scores over the subsequent 4 years in men, whereas current smoking is positively associated with changes in ADL scores over the subsequent 8 years in women [26]. These findings are consistent with our study. Another study reported that smoking in middle age increases the future risk of impaired ADL [27]. The underlying mechanisms remain unclear, necessitating further investigation.

The execution of various activities in daily life depends on cognitive function for recognition, reasoning, planning, etc. Therefore, cognitive function is also an important factor affecting ADL. A 48-month follow-up study conducted by Makino et al. in Japan found that the impairment of outdoor IADL, such as financial processing, transportation, map navigation, and shopping, was correlated with the onset of mild cognitive impairment [28], emphasizing the need for more healthcare for seniors. A study that estimated and projected trends in the number of older adults with ADL disabilities and cognitive impairments and related long-term care (LTC) costs in China over the next 20 years showed that indirect LTC costs for older adults with disabilities with cognitive impairments are expected to increase from 316 billion yuan in 2022 to 439 billion yuan in 2040, and indirect LTC costs for older adults with disabilities without cognitive impairment are projected to increase from $197 billion in 2022 to $169.7 billion in 2040 [29]. Therefore, policymakers may incorporate cognitive assessments into long-term health insurance need assessments and allocate more compensation to long-term health insurance enrollees with cognitive impairment. The results of Kashif et al.'s study show that virtual reality combined with motion imaging technology and conventional physical therapy can significantly improve motor function, balance, and ADLs in patients with Parkinson's disease [30]. Our results also suggest that good cognitive function is associated with a lower risk of impairment (BADL: *OR* = 0.68, 95% *CI*: 0.61–0.75; IADL: *OR* = 0.60, 95% *CI*: 0.54–0.67; *P* < 0.001), and attention should be paid to the cultivation and protection of cognitive function in the older groups.

Amid an aging population and longer life expectancy, the prevalence of chronic diseases, disabilities, and functional impairments among Chinese older adults has risen dramatically. An increase in the number of older adults with chronic conditions and impaired functioning, who are likely to require more healthcare services, could pose logistical and financial challenges to China's healthcare system [31]. ADL limitation is associated with aging and chronic diseases such as heart disease and diabetes, which may also be a risk factor for stroke. ADL limitation restricts activity and increases financial burdens. Older adults with ADL limitations may delay treatment for related chronic diseases, such as hypertension and diabetes, due to difficulties in accessing medical care. ADL restrictions may also increase the prevalence of depression and further increase the prevalence of stroke [32]. He et al. found that depression was correlated with ADL in the elderly and their spouses in China [33]. Yan et al.'s study showed that ADL disability and physical dysfunction were more likely to be associated with depressive symptoms among elderly people in rural China [34]. A study exploring the relationship between multimorbidity and quality of life showed that ADL, IADL, and depressive symptoms are the key mediators between multimorbidity and quality of life in older European adults, with the importance varying according to age, education, economic stress, and gender [35]. Consistent with these findings, our study also showed that depression symptoms and chronic illness significantly increased the risk of BADL and IADL impairment. Therefore, the physical and affective state of the older population deserves attention, and they should receive support and care from the government and society.

One previous study, using the trajectory of low insomnia symptoms as reference, demonstrated that low insomnia symptoms (*HR* = 1.22, 95% *CI:* 1.12–1.34), high insomnia symptoms (*HR* = 1.21, 95% *CI*: 1.05–1.41), and severe insomnia symptoms (*HR* = 1.36, 95% *CI*: 1.18–1.56) were all associated with an increased risk of ADL disability [36]. Besides, Luo et al. showed that after adjusting for potential confounders, older adults who reported shorter (≤4, 5, or 6 hours) sleep duration per night demonstrated a significantly increased risk of functional impairment compared with respondents who reported 7 hours of sleep duration per night [37]. These findings were consistent with our study. While the underlying mechanism remains unclear, several aspects may help explain it. First, insomnia can trigger a state of physiological hyperarousal, characterized by elevated blood pressure and increased cortisol levels, which can subsequently affect conditions like insulin resistance, hypertension, and coronary heart disease, and consequently contribute to functional disability. Second, sleeping deprivation can lead to musculoskeletal system impairment—lack of sleep induces inflammation and energy metabolism disorders, resulting in musculoskeletal pain and impaired tissue recovery, such as fibromyalgia, which further impacts physical function in older adults [36].

Age represents an important factor affecting ADL in older adults. The study by den Ouden et al. [38] showed that age is one of the biggest factors influencing ADL function in the elderly, through a follow-up study lasting 10 years. Due to gradual aging, the various functions of older adults will decline, which will adversely affect their ability to live independently at home and social participation, increasing the risk of ADL damage in the older population. We found that modifiable risk factors were still statistically significant in different age groups by subgroup analysis with age 45–59 as reference, and multiple risk factors were associated with a higher risk of developing BADL and IADL in the higher age group of ≥70 years old. Therefore, older adults should pay more attention to these risk factors and make appropriate adjustments.

This study holds several advantages. First, it includes a large and representative sample of 12,248 middle-aged and older adults across China. Second, multiple logistic regression models were used to adjust for potential confounders, enhancing the robustness of the findings. Reliability was further validated using PSM. By examining the associations between modified risk factors and limitations in ADL, this study provides new insights into the intervention of functional disability, offering avenues for self-intervention by older adults. With better living standards and health awareness, the modified factors covered in this study emerge as common daily-intervention activities for older adults. Targeted promotion of such activities may help reduce the prevalence of functional disability, providing a new direction for the implementation of the “Healthy China Initiative”.

However, this study also has certain limitations. As a cross-sectional study, this study could not discuss causality but only the association between modified risk factors and limitations in ADL. In future research, we plan to use longitudinal data to further explore the causal relationship between modifiable factors and limitations in ADL. Additionally, some of the data were collected through questionnaires, which may introduce subjective bias from the participants, as well as recall bias. Moreover, the number of modifiable lifestyle variables included in this study is relatively limited. Future studies should incorporate more variables (such as dietary habits, body mass index, and heavy lifting) to comprehensively investigate the impact of each modified risk factor on limitations in ADL as much as possible.

## 5. Conclusion

Modifiable risk factors are associated with limitations in ADL. As healthcare professionals, we should encourage middle-aged and older adults to actively engage in health-promoting activities, such as exercise and social participation, to prevent and mitigate the risk of functional disability. This approach not only enhances the quality of life and health outcomes but also provides valuable insights for policymakers and contributes to the goals of “Healthy China 2030”.

## Use of AI tools declaration

The authors declare they have not used Artificial Intelligence (AI) tools in the creation of this article.


