# Supplementary material for: Associations between modifiable risk factors and limitation in activities of daily living among individuals aged ≥ 45 years: Evidence from the China health and retirement longitudinal study (CHARLS)
Source: AIMS Public Health. 2025 Oct 13;12(4):1005–25. doi: 10.3934/publichealth.2025050 (PMC12795771; doi:10.3934/publichealth.2025050)
Supplement: Supplementary file 1 [file publichealth-12-04-050-s001.pdf]

Public Health

AIMS Public Health, 12(4): 1005–1025.
DOI: 10.3934/publichealth.2025050
Received: 19 May 2025
Revised: 29 August 2025
Accepted: 11 September 2025
Published: 13 October 2025

https://www.aimspress.com/journal/aimsph

Research article

Associations between modifiable risk factors and limitation in activities of daily living among individuals aged ≥ 45 years: Evidence from the China health and retirement longitudinal study (CHARLS)

Yaheng Li[1,2,†], Jian Gao[3,†], Wenzhu Song[4,†], Xiaolin Liang[5], Xinhao He[6], Fuliang Yi[7], Wenhao Song[5] and Dongliang Yin[1,*]

[1] School of Traditional Chinese Medicine, Jiangxi University of Chinese Medicine, Nanchang, Jiangxi, 330004, China
[2] Department of Clinical Laboratory, Heping Branch, Shanxi Provincial People's Hospital, Taiyuan, Shanxi, 030012, China
[3] Department of Anesthesiology, Xi'an Eighth Hospital, Xi'an, Shaanxi, 710061, China
[4] School of Public Health, Shanxi Medical University, Taiyuan, Shanxi, 030001, China
[5] Department of Geriatrics, Zhongshan Hospital of Traditional Chinese Medicine, Zhongshan, Guangdong, 528401, China
[6] School of Sports Medicine and Rehabilitation, Beijing Sport University, Beijing 100084, China
[7] Department of Public Health, Zigong Fourth People's Hospital, Zigong, Sichuan, 643000, China

* **Correspondence:** Email: dliangyin@foxmail.com.

† These authors contributed equally to this work.

Table S1. potential confounder comparison between different BADL status before and after PSM.

Variables	BADL Before PSM					BADL After PSM				
	Normal	Limited	χ^2	*P*	SMD	Normal	Limited	χ^2	*P*	SMD
Age (years), *n* (%)			280.812	<0.001	0.409			<0.001	1	<0.001
45–59	5108 (50.11)	648 (31.53)				648 (31.53)	648 (31.53)			
60–69	3268 (32.06)	781 (38.00)				781 (38.00)	781 (38.00)			
≥70	1817 (17.83)	626 (30.46)				626 (30.46)	626 (30.46)			
Gender, *n* (%)			89.215	<0.001	0.23			<0.001	1	<0.001
Female	4631 (45.43)	1168 (56.84)				1168 (56.84)	1168 (56.84)			
Male	5562 (54.57)	887 (43.16)				887 (43.16)	887 (43.16)			
Martial status, *n* (%)			43.992	<0.001	0.152			0.002	0.966	0.001
No	1073 (10.53)	321 (15.62)				322 (15.67)	321 (15.62)			
Yes	9120 (89.47)	1734 (84.38)				1733 (84.33)	1734 (84.38)			
Residence, *n* (%)			37.478	<0.001	0.152			0.001	0.971	0.001
Non-rural	3113 (30.54)	489 (23.80)				490 (23.84)	489 (23.80)			
Rural	7080 (69.46)	1566 (76.20)				1565 (76.16)	1566 (76.20)			
Educational levels, *n* (%)			199.788	<0.001	0.345			0.003	1	0.002
No formal education	2699 (26.48)	824 (40.10)				824 (40.10)	824 (40.10)			
Elementary education	2592 (25.43)	547 (26.62)				547 (26.62)	547 (26.62)			
Secondary education	3040 (29.82)	463 (22.53)				464 (22.58)	463 (22.53)			
Higher education	1862 (18.27)	221 (10.75)				220 (10.71)	221 (10.75)			

Note: SMD: standardized mean difference.

Table S2. potential confounder comparison between different IADL status before and after PSM.

Variables	IADL Before PSM					IADL After PSM				
	Normal	Limited	χ^2	*P*	SMD	Normal	Limited	χ^2	*P*	SMD
Age (years), *n* (%)			245.154	<0.001	0.393			0.002	0.999	0.001
45–59	5159 (49.56)	597 (32.48)				597 (32.48)	597 (32.48)			
60–69	3385 (32.52)	664 (36.13)				665 (36.18)	664 (36.13)			
≥70	1866 (17.93)	577 (31.39)				576 (31.34)	577 (31.39)			
Sex, *n* (%)			99.421	<0.001	0.254			<0.001	1	<0.001
Female	4732 (45.46)	1067 (58.05)				1067 (58.05)	1067 (58.05)			
Male	5678 (54.54)	771 (41.95)				771 (41.95)	771 (41.95)			
Martial status [*n* (%)]			47.827	<0.001	0.164			<0.001	1	<0.001
No	1098 (10.55)	296 (16.10)				296 (16.10)	296 (16.10)			
Yes	9312 (89.45)	1542 (83.90)				1542 (83.90)	1542 (83.90)			
Residence, *n* (%)			26.403	<0.001	0.133			<0.001	1	<0.001
Non-rural	3154 (30.30)	448 (24.37)				448 (24.37)	448 (24.37)			
Rural	7256 (69.70)	1390 (75.63)				1390 (75.63)	1390 (75.63)			
Educational levels, *n* (%)			233.315	<0.001	0.4			0.004	1	0.002
No formal education	2767 (26.58)	756 (41.13)				756 (41.13)	756 (41.13)			
Elementary education	2631 (25.27)	508 (27.64)				508 (27.64)	508 (27.64)			
Secondary education	3090 (29.68)	413 (22.47)				414 (22.52)	413 (22.47)			
Higher education	1922 (18.46)	161 (8.76)				160 (8.71)	161 (8.76)			

Note: SMD: standardized mean difference.

AIMS Press

© 2025 the Author(s), licensee AIMS Press. This is an open access article distributed under the terms of the Creative Commons Attribution License (https://creativecommons.org/licenses/by/4.0)
